# Vamorolone improves Becker muscular dystrophy and increases dystrophin protein in *bmx* model mice

**DOI:** 10.1016/j.isci.2023.107161

**Published:** 2023-06-16

**Authors:** Nikki M. McCormack, Nhu Y. Nguyen, Christopher B. Tully, Trinitee Oliver, Alyson A. Fiorillo, Christopher R. Heier

**Affiliations:** 1 Center for Genetic Medicine Research, Children’s National Hospital, Washington, DC, USA; 2 Department of Biology, Howard University, Washington, DC, USA; 3 Department of Genomics and Precision Medicine, The George Washington University, Washington, DC, USA

**Keywords:** Pharmacology, Biological sciences, Neuroscience

## Abstract

There is no approved therapy for Becker muscular dystrophy (BMD), a genetic muscle disease caused by in-frame dystrophin deletions. We previously developed the dissociative corticosteroid vamorolone for treatment of the allelic, dystrophin-null disease Duchenne muscular dystrophy. We hypothesize vamorolone can treat BMD by safely reducing inflammatory signaling in muscle and through a novel mechanism of increasing dystrophin protein via suppression of dystrophin-targeting miRNAs. Here, we test this in the *bmx* mouse model of BMD. Daily oral treatment with vamorolone or prednisolone improves *bmx* grip strength and hang time phenotypes. Both drugs reduce myofiber size and decrease the percentage of centrally nucleated fibers. Vamorolone shows improved safety versus prednisolone by avoiding or reducing key side effects to behavior and growth. Intriguingly, vamorolone increases dystrophin protein in both heart and skeletal muscle. These data indicate that vamorolone, nearing approval for Duchenne, shows efficacy in *bmx* mice and therefore warrants clinical investigation in BMD.

## Highlights

• Vamorolone and prednisone improve muscle strength and pathology in BMD model mice
• Vamorolone shows an improved safety profile versus prednisone
• Anti-inflammatory steroidal drugs increase dystrophin protein in muscle and heart
• Dystrophin protein levels inversely correlate with dystrophin-targeting miRNAs

## Graphical abstract

**Figure:**
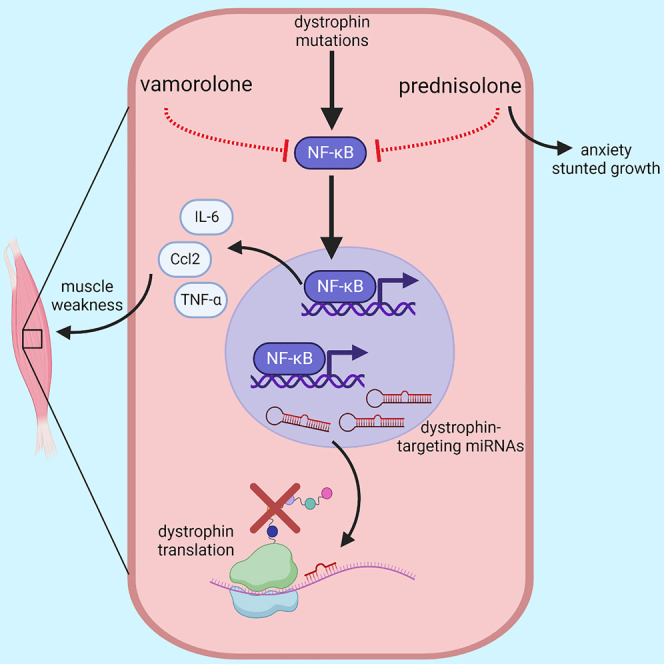

## Introduction

Becker muscular dystrophy (BMD) is a variably debilitating muscle disease caused by in-frame dystrophin mutations resulting in expression of truncated dystrophin isoforms that are often expressed at reduced levels.^1^^,^^2^^,^^3^ Patients with BMD have a wide variety of disease severity ranging from asymptomatic to as severe as Duchenne muscular dystrophy (DMD)—a disease primarily caused by out-of-frame dystrophin mutations resulting in complete loss of dystrophin. Many patients with BMD have motor impairments and up to 50% of patients with BMD die from cardiomyopathy.^4^^,^^5^^,^^6^^,^^7^^,^^8^ Drug development for BMD has been hindered by the previous lack of a mouse model, and there are currently only two drugs in BMD clinical trials in comparison to approximately 30 drugs in clinical trials for DMD. There is currently no approved treatment for BMD.

The standard of care for DMD is chronic administration of glucocorticoids, such as prednisone and deflazacort, which slow disease progression by suppressing inflammation through inhibition of NF-κB signaling.^9^ However, chronic administration of traditional glucocorticoids also activates pathways that cause adverse side effects including stunted growth, behavior issues, diabetes, bone loss, excessive weight gain, and immunosuppression. Traditional glucocorticoids are rarely prescribed to patients with BMD due to their harsh side effects and unclear efficacy.^10^ It is unknown how corticosteroids affect BMD pathology and function.

We previously demonstrated that the first-in-class dissociative corticosteroid vamorolone—a dual action drug that is a selective glucocorticoid receptor (GR) agonist as well as a mineralocorticoid receptor (MR) antagonist—improves disease outcomes in *mdx* mice while bypassing GR-transactivation side effect pathways that are activated by traditional glucocorticoids.^11^ In addition to acting as a GR agonist, we found that vamorolone acts as an MR antagonist and improves *mdx* heart health whereas prednisolone, an MR agonist, worsens cardiac fibrosis and function.^12^ In contrast to traditional glucocorticoids, the improved safety profile and cardioprotective properties of vamorolone suggest that it may provide a safe and effective treatment for patients with BMD. In a randomized, double-blind, placebo- and prednisone-controlled clinical trial of vamorolone in DMD, vamorolone showed equal efficacy to prednisone while reducing corticosteroid-associated safety concerns, such as stunting of growth (NCT03439670).^13^

Dystrophin-targeting microRNAs (DTMs) bind to the dystrophin 3′ untranslated region (UTR) of transcripts and downregulate protein expression.^14^ The expression of DTMs such as miR-146a is increased by inflammatory NF-κB signaling, and this leads to upregulation of DTMs in muscle diseases with an inflammatory component such as BMD, DMD, and myositis.^14^^,^^15^^,^^16^ Consequently, anti-inflammatory drugs such as prednisolone and vamorolone reduce expression of DTMs in *mdx* mice.^14^^,^^15^^,^^16^ Similar expression increases and anti-inflammatory drug responses for miR-146a are conserved in humans with other inflammatory diseases, such as inflammatory bowel disease.^17^^,^^18^ These data suggest that vamorolone may also be beneficial for BMD by increasing dystrophin levels through suppression of DTMs.

The *bmx* mouse provides the first preclinical mouse model of BMD featuring CRISPR-induced deletion of endogenous dystrophin exons 45–47, which models the most common BMD patient mutation and is associated with more severe skeletal muscle pathology and cardiac outcomes.^19^^,^^20^^,^^21^ Vamorolone, developed for clinical use through preclinical trials in *mdx* mice, provides a drug that could safely treat chronic inflammatory signaling, muscle pathology, weakness, cardiomyopathy, and low dystrophin levels in BMD. Here, we investigate impacts of vamorolone versus the traditional glucocorticoid prednisolone on disease in *bmx* mice. Untreated, the *bmx* mouse has phenotypes intermediate to wild type (WT) and *mdx52* mice.^19^ Here, we find daily oral vamorolone increases *bmx* motor function, improves skeletal muscle pathology, and decreases pathological gene expression. Vamorolone avoids side effects of chronic prednisolone treatment including anxiety and stunted growth. We find that vamorolone also increases dystrophin protein and reduces DTM expression in both cardiac and skeletal muscle. Our data provide the first *bmx* preclinical trial supporting BMD drug development and give new insights into the potential for dissociative steroids such as vamorolone to more broadly treat muscle diseases with chronic inflammation, cardiomyopathy, alternative dystrophin isoforms, or reduced dystrophin.

## Results

### Vamorolone safely improves motor function of *bmx* mice

We began by investigating the consequences of vamorolone on dystrophic mouse strength and behavior. In their natural history, *bmx* mice have impaired motor function intermediate to WT and *mdx* by 10 weeks of age.^19^ Here, starting at 6 weeks of age, randomized and blinded treatment groups of *bmx* mice were treated with vehicle, vamorolone (20 mg/kg/d), or prednisolone (5 mg/kg/d), using daily oral administration via ingestion of cherry syrup formulations (n = 12 per group). These doses were chosen based upon our extensive experience in the *mdx* mouse model of DMD. We used prednisolone as it is the active form of prednisone, and have found 5 mg/kg prednisolone to be safe and effective in *mdx* mice.^11^ We have shown doses of 5–45 mg/kg vamorolone are well tolerated by *mdx* mice, and use 20 mg/kg vamorolone here as we previously established this dose to be safe and effective at treating both *mdx* muscle and heart.^12^

**Figure 1:**
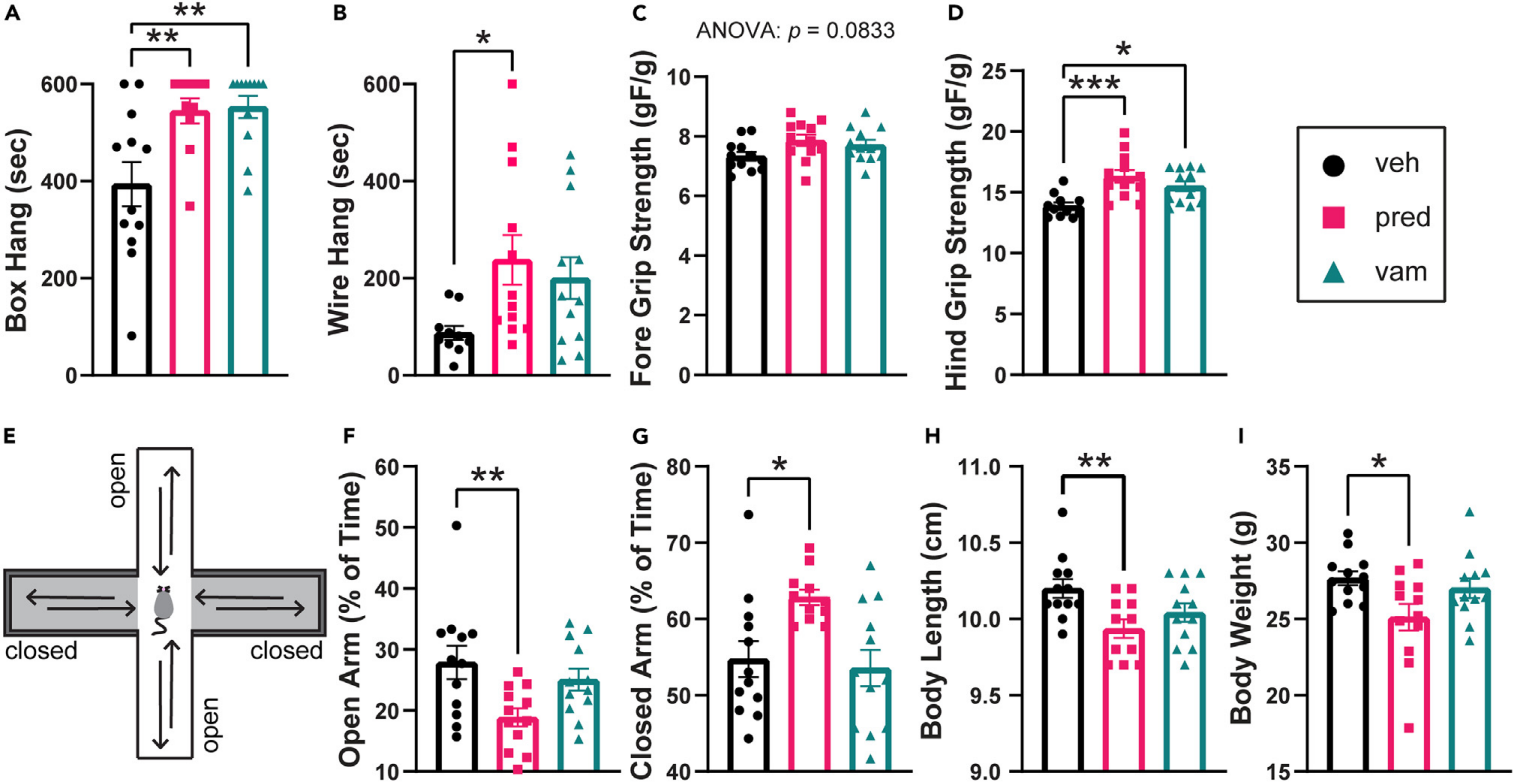
Vamorolone safely improves strength of *bmx* mice Mice were treated with daily oral vehicle (veh; cherry syrup), vamorolone (vam; 20 mg/kg/d), or prednisolone (pred; 5 mg/kg/d) beginning at 6 weeks of age. (A) Prednisolone and vamorolone significantly increased hang time in the box hang test. (B) Prednisolone significantly increased hang time in the wire hang test while hang time was moderately increased with vamorolone. (C and D) Prednisolone and vamorolone had no effect on forelimb grip strength but significantly increased hindlimb grip strength. n = 12 mice per group. (E) Diagram of the elevated plus maze. The maze consists of two open arms (no walls) and two protected, closed arms (enclosed by walls). Mice are placed in the center of the maze and allowed to explore for 5 minutes. (F and G) In the elevated plus maze, prednisolone-treated mice spent significantly less time in the open arms and significantly more time in the closed arms whereas vamorolone-treated mice had no significant changes in time spent in open or closed arms. n = 12 per group. (H and I) Prednisolone-treated mice had significantly reduced body length and weight. Body weight and length are unchanged with vamorolone treatment. n = 12 per group. Data are analyzed by one-way ANOVA followed by *post hoc* Holm-Sidak’s multiple comparisons test. Data are represented as mean ± S.E.M. ∗p ≤ 0.05, ∗∗p ≤ 0.01, ∗∗∗p ≤ 0.001.

Both prednisolone (38.4% increase, p = 0.0041) and vamorolone (40.5% increase, p = 0.0035) showed efficacy through significantly increased mouse strength as assayed by suspension time in the four-limb box hang test (Figure 1A). Two vehicle-treated mice were able to hang for the full 10 min of this test, at least seven mice per drug group were able to hang for the full 10 min. Suspension time in the two-limb wire hang test was also significantly increased with prednisolone (172.9% increase, p = 0.0348) and was modestly increased in the vamorolone-treated (129.6% increase, p = 0.0694) group (Figure 1B). Assaying forelimb grip strength, both drugs showed modest improvements that did not reach significance (Figure 1C). For hindlimb grip strength, both prednisolone (17.5% increase, p = 0.0007) and vamorolone (11.7% increase, p = 0.0117) significantly increased the strength of BMD model mice (Figure 1D). These effects are consistent with therapeutic efficacy of vamorolone and prednisolone on *bmx* muscle strength phenotypes.

Anxiety behavior in the elevated plus maze, along with stunted growth, were assayed as measures of drug safety.^11^ To assess anxiety behavior, vehicle- and drug-treated *bmx* mice were placed on the elevated plus maze and their movement was recorded for 5 min. The elevated plus maze consists of two open arms without walls and two closed arms that are enclosed by walls.^22^ Mice that are anxious will avoid spending time in the open arms and spend more time in the protected, closed arms of the maze whereas non-anxious mice will explore both open and closed arms (Figure 1E). Prednisolone-treated mice spent significantly more time in the safety of the closed arms (14.8% increase, p = 0.02) and significantly less time (32.4% decrease, p = 0.0084) in the exposed, open arms of the plus maze in comparison to vehicle-treated mice (Figures 1F and 1G). In contrast, the time spent in the open and closed arms did not differ between vehicle-treated and vamorolone-treated mice (Figures 1F and 1G). This is consistent with prednisolone causing increased anxiety, while vamorolone avoids this effect. At the conclusion of the trial, we also found that prednisolone significantly reduced body length (2.6% decrease, p = 0.0092) and body weight (9.3% decrease, p = 0.023; Figures 1H and 1I), consistent with stunted growth. In a previous study, prednisolone treatment of *mdx* mice at an earlier age (treated from 2 to 8 weeks) resulted in a larger 6.3% decrease in body length.^11^ Vamorolone-treated *bmx* mice did not show a significant difference in body length, consistent with prior studies in *mdx* mice treated with up to 45 mg/kg vamorolone from 2 to 8 weeks. These data are consistent with an improved safety profile for the dissociative steroid vamorolone in comparison to traditional glucocorticoids in patients with DMD.^23^

### Vamorolone reduces skeletal muscle pseudohypertrophy and pathology

**Figure 2:**
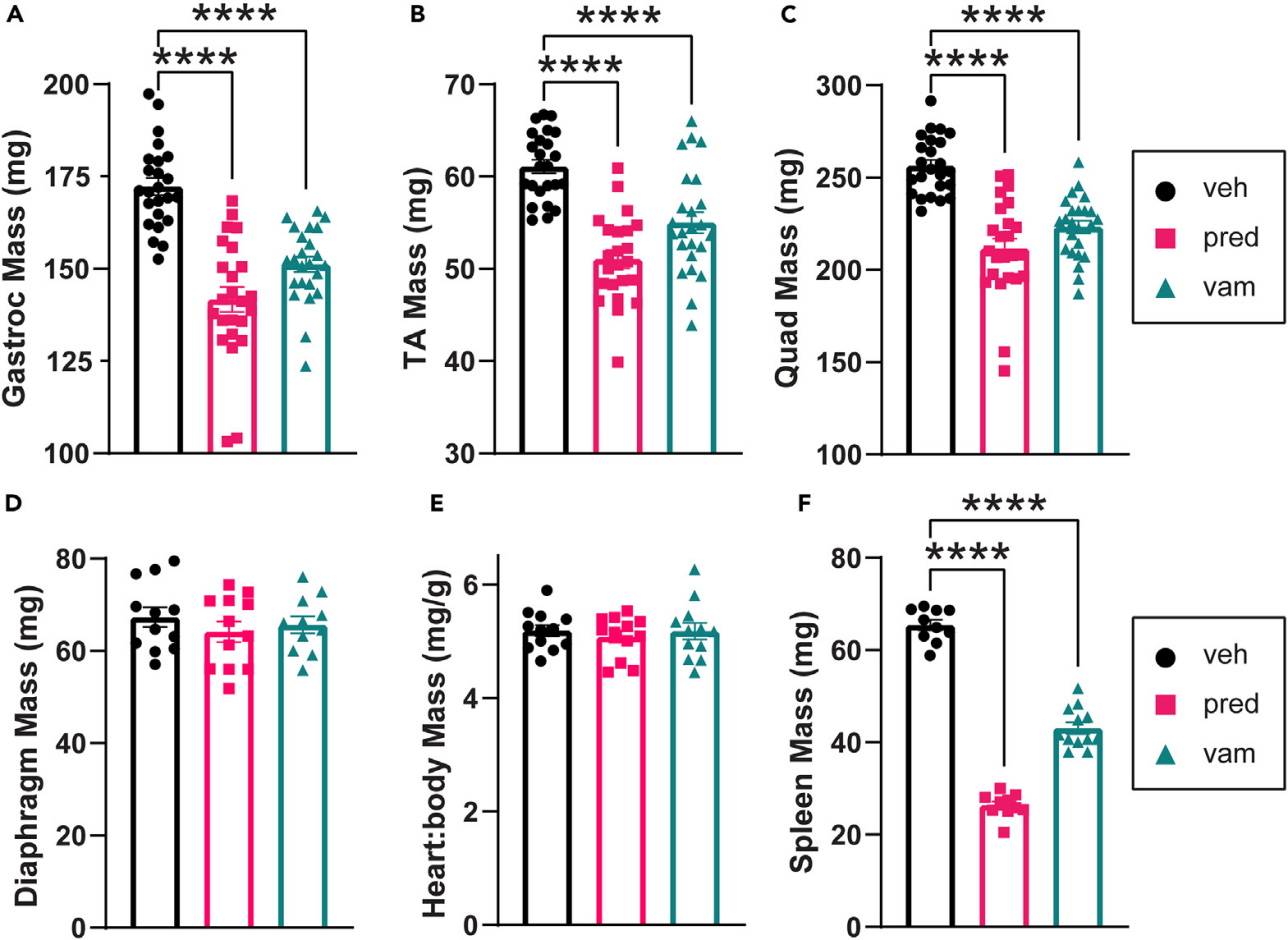
Treatments reduce *bmx* muscle pseudohypertrophy (A–C) Prednisolone and vamorolone reduce mass of gastrocnemius, tibialis anterior, and quadriceps. n = 24 per group. (D) Prednisolone and vamorolone did not change mass of the diaphragm. n = 12 per group. (E) Heart:body mass is not significantly impacted by prednisolone and vamorolone treatment. (F) Spleen mass is significantly reduced with prednisolone and vamorolone treatment. n = 12 per group. Data are analyzed by one-way ANOVA followed by *post hoc* Holm-Sidak’s multiple comparisons test. Data are represented as mean ± S.E.M. ∗p ≤ 0.05, ∗∗p ≤ 0.01, ∗∗∗∗p ≤ 0.0001.

After 10 weeks of treatment, we assayed tissue weights and performed terminal endpoint measures. Patients with BMD characteristically develop enlarged calf muscles even though these muscles are weaker.^24^ This is termed pseudohypertrophy and it also occurs in both patients with DMD and *mdx* mice. Such pseudohypertrophy is the result of increases in non-contractile tissue and in myofiber branching, with non-contractile tissue including a combination of inflammation, degenerating/regenerating tissue, fibrosis, and fatty tissue infiltration.^25^^,^^26^^,^^27^^,^^28^^,^^29^^,^^30^ Previously, we showed that *bmx* mice develop muscle pseudohypertrophy consistent with BMD and enlarged spleens consistent with their chronic inflammatory disease state.^19^ Here, we find that prednisolone and vamorolone significantly reduced skeletal muscle pseudohypertrophy in the gastrocnemius, tibialis anterior (TA), and quadriceps (Figures 2A–2C). We observed no significant effect on diaphragm or heart mass (Figures 2D and 2E). Both treatments significantly reduced spleen mass (Figure 2F). These changes are consistent with efficacy in treating dystrophic muscle and inflammation in BMD model mice.

**Figure 3:**
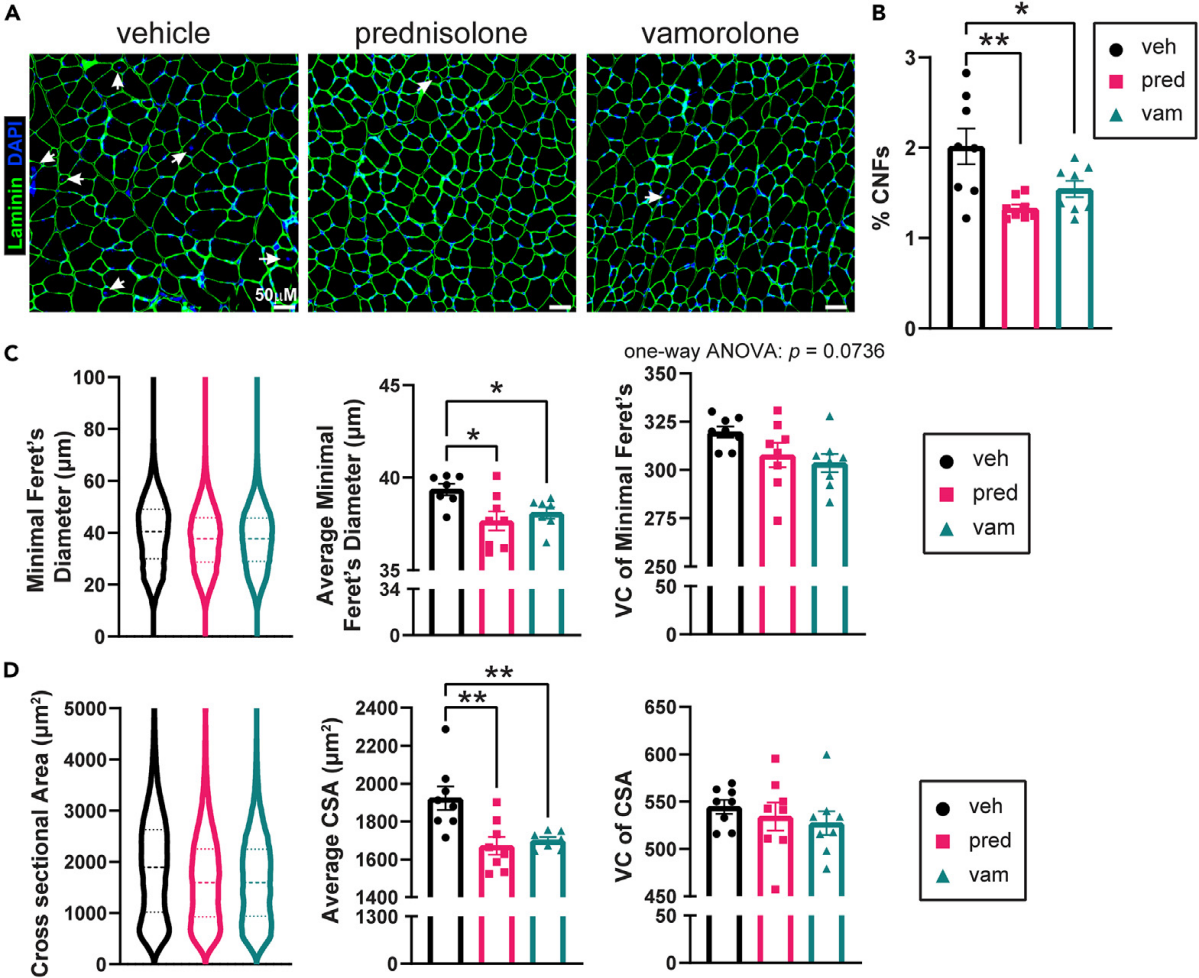
Treatment reduces myofiber pathology (A) Gastrocnemius muscle cross-sections were stained with laminin and DAPI to visualize myofiber membranes and nuclei. White arrows denote centrally nucleated fibers (CNFs). Scale bar = 50 μm. (B) The percentage of CNFs was significantly reduced with prednisolone and vamorolone. (C) Minimal Feret’s diameter was determined for each myofiber using MuscleJ and the variance coefficient (VC) was calculated. Average minimal Feret’s diameter is significantly reduced with prednisolone and vamorolone. Variability of the minimal Feret’s diameter was modestly reduced with vamorolone. (D) Cross-sectional area (CSA) was determined for each myofiber using MuscleJ and the VC was calculated. Average CSA was significantly reduced with prednisolone and vamorolone. The VC of the CSA was unchanged with prednisolone and vamorolone treatment. n = 8 per group. Data are analyzed by one-way ANOVA followed by *post hoc* Holm-Sidak’s multiple comparisons test. Data are represented as mean ± S.E.M. ∗p ≤ 0.05, ∗∗p ≤ 0.01. See also Figure S1.

We next assessed myofiber histopathology given that *bmx* mice have increased percentage of centrally nucleated fibers (CNFs) and myofiber size variability as a result of disease.^19^ Examining gastrocnemius muscle, both vamorolone and prednisolone treatment resulted in significantly fewer CNFs in *bmx* mice (Figures 3A and 3B). Myofibers in the gastrocnemius also showed a significantly reduced average minimal Feret’s diameter and average myofiber cross-sectional area (CSA) and showed a trend of decrease in fiber size variability for each (Figures 3C and 3D). In the TA, we found that both drugs caused significant decreases in the average minimal Feret’s diameter and average CSA, and vamorolone significantly decreased variability in the minimal Feret’s diameter of fibers (Figure S1). Consistent with impacts on calf pseudohypertrophy, which is a key phenotype of patients with BMD, these data demonstrate efficacy in treating *bmx* muscle pathology.^5^^,^^24^^,^^31^

### Vamorolone reduces pathological gene expression in *bmx* skeletal muscle

In addition to muscle weakness and myofiber phenotypes, the *bmx* mouse shows an increase in infiltrating immune cells and increased expression of inflammatory genes and microRNAs consistent with chronic inflammatory disease.^19^ Here, analysis of hematoxylin and eosin-stained gastrocnemius and TA cross-sections showed no differences in the percent area of necrosis and inflammation (Figures S2A, S2B, S3A, and S3B).

**Figure 4:**
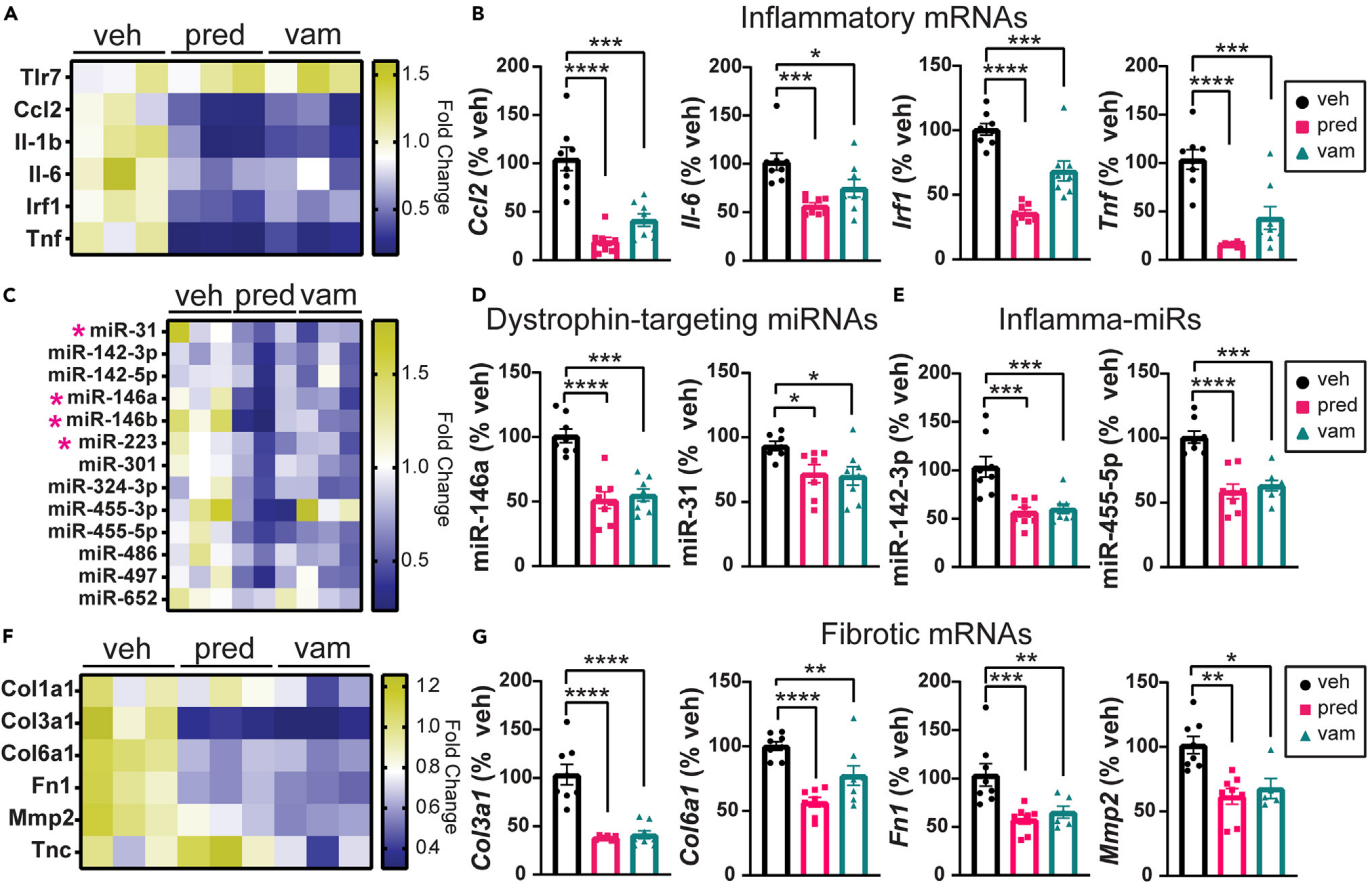
Treatments reduce pathological gene expression in *bmx* skeletal muscle Markers of inflammation and fibrosis were assayed via qRT-PCR in gastrocnemius muscle of treated mice. (A) Heatmap of inflammatory gene expression in gastrocnemius muscle. (B) Graphs showing expression of inflammatory mRNAs in gastrocnemius muscle. Expression of *Ccl2*, *Il-6*, *Irf1*, and *Tnf* is significantly reduced with prednisolone and vamorolone treatment. (C) Heatmap of dystrophin-targeting miRNAs (DTMs) and inflamma-miRs. Pink asterisks denote DTMs. (D and E) Graphs showing expression of DTMs and inflammatory miRNAs in gastrocnemius muscle. Expression of miR-146a, miR-223, miR-142-3p, and miR-455-5p is significantly reduced with prednisolone and vamorolone treatment. (F) Heatmap of fibrotic gene expression in gastrocnemius muscle. (G) Graphs showing expression of fibrotic genes. Expression of *Col3a1*, *Col6a1*, *Fn1*, and *Mmp2* is significantly reduced with prednisolone and vamorolone treatment. n = 8 per group. Data are analyzed by one-way ANOVA followed by *post hoc* Holm-Sidak’s multiple comparisons test. Data are represented as mean ± S.E.M. ∗p ≤ 0.05, ∗∗p ≤ 0.01, ∗∗∗p ≤ 0.001, ∗∗∗∗p ≤ 0.0001. See also Figures S2–S4.

We next examined a panel of inflammatory mRNAs and miRNAs. Both prednisolone and vamorolone treatment significantly reduced the expression of inflammatory genes including *Ccl2*, *Il-6*, *Irf1*, and *Tnf* in *bmx* gastrocnemius muscle (Figures 4A and 4B). Similar effects were observed in the TA, in which inflammatory genes were reduced with both prednisolone and vamorolone (Figures S3C and S3D). Consistent with mRNA, the expression of miRNAs upregulated by inflammatory disease was also significantly reduced with both prednisolone and vamorolone treatment in *bmx* gastrocnemius (Figures 4C–4E). Levels of DTMs, including miR-146a and miR-31, are significantly reduced in the gastrocnemius with prednisolone and vamorolone treatment (Figures 4C and 4D).

In the TA, levels of miR-146a and miR-223 are significantly reduced with prednisolone while none of the DTMs are reduced with vamorolone in the TA (Figures S3E and S3F). Levels of miR-142-3p and miR-142-5p are unchanged with prednisolone and vamorolone in the TA (Figures S3E and S3F). miR-455-5p levels are significantly reduced with prednisolone and modestly reduced with vamorolone while miR-497 is significantly reduced with both treatments (Figures S3E and S3F).

Muscle from *bmx* mice shows elevated expression of fibrotic genes.^19^ Both vamorolone and prednisolone treatment cause significant reductions in fibrosis gene expression in gastrocnemius muscle, including *Col3a1*, *Col6a1*, *Fn1*, and *Mmp2* (Figures 4F and 4G). Similar effects were observed in TA muscle (Figures S4H and S4I).

### Vamorolone reduces markers of inflammation and fibrosis in the heart

**Figure 5:**
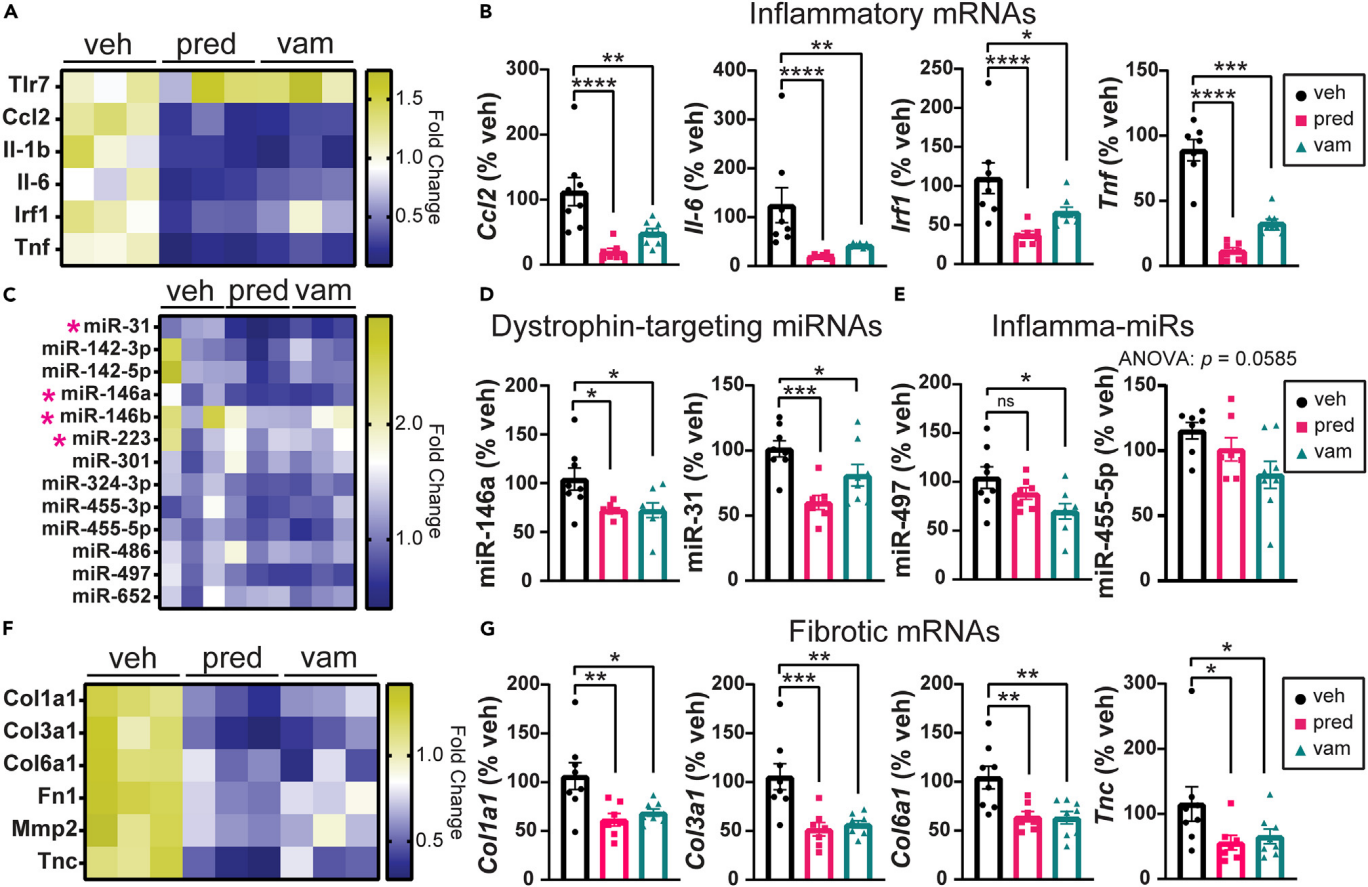
Treatment reduces pathological gene expression in *bmx* hearts (A) Heatmap of inflammatory gene expression in the heart. (B) Graphs showing expression of inflammatory mRNAs in the heart. Expression of *Ccl2*, *Il-6*, *Irf1*, and *Tnf* is significantly reduced with prednisolone and vamorolone treatment. (C) Heatmap of dystrophin-targeting miRNAs (DTMs) and inflamma-miRs in the heart. Pink asterisks denote DTMs. (D) Expression of miR-146a and miR-31 is significantly reduced with prednisolone and vamorolone treatment. (E) Expression of miR-497 is significantly reduced with vamorolone but not prednisolone treatment. Expression of miR-455-5p is moderately reduced with vamorolone treatment. (F) Heatmap of fibrotic gene expression in the heart. (G) Graphs showing expression of fibrotic genes. Expression of *Col1a1*, *Col3a1*, *Col6a1*, and *Tnc* is significantly reduced in the heart with prednisolone and vamorolone treatment. n = 8 per group. Data are analyzed by one-way ANOVA followed by *post hoc* Holm-Sidak’s multiple comparisons test. Data are represented as mean ± S.E.M. ∗p ≤ 0.05, ∗∗p ≤ 0.01, ∗∗∗p ≤ 0.001, ∗∗∗∗p ≤ 0.0001.

Previously, we showed that *bmx* hearts have increased expression of inflammatory and fibrotic genes.^19^ Here, we found both prednisolone and vamorolone significantly reduce expression of many inflammatory genes including *Ccl2*, *Il-6*, *Irf1*, and *Tnf* (Figures 5A and 5B). Both treatments significantly reduce levels of the DTMs miR-146a and miR-31 (Figures 5C and 5D). Levels of miR-497 and miR-455 were significantly reduced with vamorolone but not prednisolone (Figures 5C and 5E). Prednisolone and vamorolone also significantly reduce expression of many fibrotic genes in the heart including *Col1a1*, *Col3a1*, *Col6a1*, and *Tnc* (Figures 5F and 5G). We next stained heart cross-sections with Sirius red fast green to visualize and quantify fibrosis. We found no clear signs of fibrosis at this age in any treatment group of *bmx* mice and no statistical differences in the percentage of fibrotic staining area with prednisolone or vamorolone treatment (Figure S4A). We also stained heart cross-sections with IgM to assess heart damage and found no IgM-positive staining in any mouse (Figure S4B). Together, these data are consistent with both drugs effectively targeting pre-symptomatic gene expression changes characteristic of *bmx* cardiomyopathy.

### Vamorolone increases dystrophin protein levels

**Figure 6:**
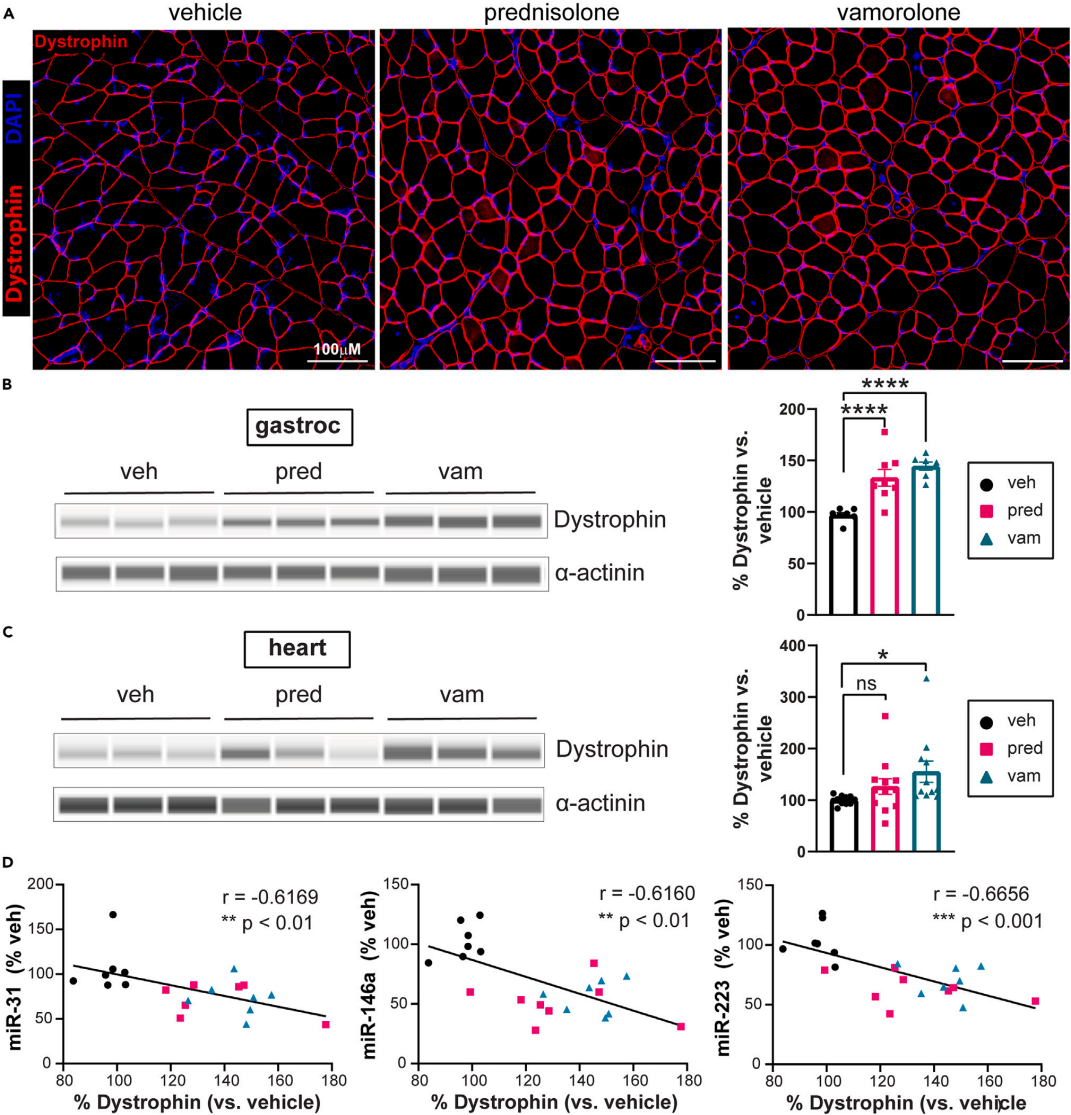
Vamorolone increases dystrophin protein in *bmx* heart and skeletal muscle Dystrophin protein levels were assayed in muscle and heart via immunofluorescence and quantified by Wes capillary-based electrophoresis. (A) Representative images of gastrocnemius cross-sections immunostained with dystrophin. Scale bar = 100 μm. (B) Dystrophin protein levels are significantly increased in the gastrocnemius with prednisolone and vamorolone. n = 8 per group. (C) Dystrophin protein levels are slightly elevated in the heart with prednisolone and are significantly increased with vamorolone. n = 12 per group. (D) Dystrophin protein levels in the gastrocnemius are inversely correlated with expression of miR-31, miR-146a, and miR-223. Data are analyzed by one-way ANOVA followed by *post hoc* Holm-Sidak’s multiple comparisons test. Data are represented as mean ± S.E.M. ns p > 0.05, ∗p ≤ 0.05, ∗∗∗∗p ≤ 0.0001. See also Figure S5.

Having found reduced expression of the DTMs miR-146a and miR-31, we next examined dystrophin protein levels. Immunofluorescence staining showed that both drugs caused increases in dystrophin protein in the gastrocnemius (Figure 6A). Using Wes capillary electrophoresis to quantify dystrophin protein, we found that both drugs significantly increase dystrophin protein. Dystrophin increases of 49% (p < 0.0001) were found in the gastrocnemius and 55% (p = 0.0173) in the heart with vamorolone treatment (Figures 6B and 6C). Dystrophin increases of 37% (p < 0.0001) were found in the gastrocnemius and 26% (p = 0.2163) in the heart with prednisolone treatment. Supporting an inverse relationship between DTM and dystrophin protein levels, we observed a significant inverse correlation (p < 0.01) between dystrophin protein levels and the expression of three DTMs (miR-31: r = −0.6169; miR-146a: r = −0.6160; miR-223: r = −0.6656) which decreased in response to drug treatment within the gastrocnemius muscle (Figure 6D). Also consistent with the link between DTMs and dystrophin protein, in a different muscle where we observed a reduced impact of drug treatment on DTMs, we also saw no significant impact on dystrophin levels (TA, Figures S5A–S5C). Together, these data are consistent with DTM regulation of dystrophin protein levels during disease and support therapeutic induction of dystrophin protein as a novel therapeutic mechanism for vamorolone in BMD and as a DMD exon-skipping co-therapy.

## Discussion

We find both vamorolone and prednisolone improve muscle disease in BMD in a manner that also increases dystrophin protein through effects on inflammatory miRNA pathways. Vamorolone is a first-in-class dissociative steroid which provides selective anti-inflammatory efficacy through the GR, with improved safety versus prednisone via reduced effects on steroid transactivation pathways. Impacts of corticosteroids on inflammatory miRNAs show an inverse correlation with dystrophin protein levels. Additionally, vamorolone acts through the evolutionarily conserved MR receptor as an antagonist to protect hearts against dystrophic cardiomyopathy. This property is analogous to the specific antagonist eplerenone, and acts in direct contrast to prednisolone which is a MR receptor agonist. Together, these three properties make vamorolone an attractive therapeutic for BMD and inform future drug development for a broader group of conditions sharing muscle, heart, and immune-mediated pathology.

Until this point, the development of BMD therapeutics has been hindered by a lack of animal models. While some patients are treated symptomatically for cardiomyopathy with angiotensin-converting enzyme inhibitors, there are currently no approved treatments for BMD. Weekly prednisone treatment is currently being tested in a combination of patients with BMD and limb girdle muscular dystrophy (LGMD) (NCT04054375). Two additional drugs are currently in BMD-focused clinical trials. The first is EDG-5506, a fast skeletal muscle myosin inhibitor (NCT05160415). The other is daily vamorolone, for which we here provide the first *in vivo* evidence in a mouse model of BMD (NCT05166109). In contrast to the small number of interventional trials for BMD, there are currently approximately 30 interventional trials ongoing for DMD. Moving forward, it will be increasingly important to develop BMD therapeutics because the goal of many DMD therapeutics is to convert a severe DMD genotype (dystrophin-null) into a milder BMD-like phenotype (in-frame, reduced dystrophin) through exon skipping or gene therapies.

Our preclinical data also support the efficacy of prednisone in treating BMD, for which it can immediately be prescribed. We find prednisolone improves muscle strength and pathology in BMD model mice, reduces dystrophin-targeting miRNAs, and increases dystrophin levels. To help manage side effects of prednisone, here we delay treatment of mice to start at 6 weeks as opposed to the 2-week initiation point in our prior studies in the more severe *mdx* model of DMD. Doing this results in a smaller percentage of growth inhibition by prednisone for *bmx* mice (2.6%) in comparison to *mdx* mice (6.3%).^19^ Many boys with DMD are currently prescribed chronic glucocorticoids starting around four to seven years of age, which causes developmental delays including growth stunting, delayed skeletal maturation, and delayed puberty.^32^^,^^33^^,^^34^ These side effects can be reduced or avoided in patients with BMD in comparison to those with DMD by delaying the initiation of treatment to later ages, perhaps in teenage or post-puberty years. However, some negative effects of chronic glucocorticoids are not developmentally restricted and would still affect quality of life. An alternative strategy to help reduce these impacts may be to apply intermittent or weekend dosing regimens such as in the ongoing weekly prednisone dosing trial in patients with BMD and LGMD (NCT04054375). However, data from a recent trial in DMD support the use of daily corticosteroid regimens as opposed to intermittent regimens, so further investigations into alternative regimens such as these are needed.^35^

Previous studies in our lab show DTMs bind to the dystrophin 3′ UTR of transcripts and downregulate protein expression of reporter constructs (luciferase) with the human dystrophin 3′ UTR.^14^ We find that 1) expression of DTMs is increased by inflammatory NF-κB signaling, 2) these DTMs are upregulated in muscle diseases such as BMD, DMD, and myositis, and 3) vamorolone reduces expression of DTMs in *mdx* mice.^14^^,^^15^^,^^16^ Given that *mdx* mice do not produce dystrophin protein, we were unable to examine dystrophin protein levels in previous studies. Here, we find vamorolone significantly reduces DTMs and significantly increases dystrophin protein in both skeletal muscle and the heart. Consistent with this, previous studies show increased levels of dystrophin in *mdx* treated with exon-skipping oligonucleotides and prednisolone in comparison to *mdx* mice treated only with exon-skipping oligonucleotides.^36^ In BMD patient muscle cultures, treatment with methylprednisolone increased dystrophin expression.^37^ Interestingly, wild-type iPSC-derived myofibers treated with prednisolone have increased dystrophin suggesting that even suppression of basal levels of DTMs may be sufficient to increase dystrophin.^38^ We find that vamorolone significantly increases dystrophin in the gastrocnemius and heart but not the TA with vamorolone treatment. We also show that with vamorolone treatment, the DTMs miR-146a and miR-31 are significantly reduced in the gastrocnemius and heart but not in the TA. Together, these data suggest that reduction of miR-146a and miR-31 may be responsible for increased dystrophin protein levels. Future studies examining knockout of DTMs or miRNA inhibitors in *bmx* or in *mdx* mice treated with exon-skipping oligonucleotides are needed to directly link dystrophin protein levels and expression of DTMs. The differential involvement of muscles seen by miRs and dystrophin increase may be related to differential severity of muscle involvement seen in human MRI studies where the TA shows slower progression than the gastrocnemius.^39^

In *mdx* mice, we find in previous studies that loss of dystrophin causes a “second hit” which makes hearts susceptible to MR-driven heart damage whereas MR agonists have no effect on wild-type mice with full-length dystrophin.^12^ Here, we find that both prednisolone and vamorolone significantly reduce expression of inflammatory and fibrotic genes. The dual GR/MR agonist prednisolone also does not increase cardiac fibrosis or damage in *bmx* mice. This suggests the dystrophinΔex45-47 may provide some level of protection for the heart from MR-mediated damage and fibrosis. However, further investigations with aged mice, stressed hearts, alternative dystrophin isoforms, or MR-specific ligands will be needed to more fully gauge what levels of protection are provided.

An important question is whether prednisone’s efficacy in DMD comes from anti-inflammatory effects in immune cell types or directly within diseased myofibers. We find that prednisolone and vamorolone significantly reduce levels of inflammatory genes and miRNAs in skeletal muscle. However, hematoxylin and eosin staining of skeletal muscles did not reveal any differences in immune cell infiltration in drug-treated mice. This suggests that prednisolone and vamorolone are acting directly on myofibers to reduce inflammatory gene expression within muscle fibers and myonuclei. Consistent with this, prednisolone improves the disease phenotype of iPSC-derived myofibers with DMD patient mutations.^38^

While prednisolone and vamorolone ameliorate disease pathology in *bmx* mice, vamorolone avoids or reduces side effects of prednisolone that negatively impact quality of life. Previous preclinical reports show that vamorolone has reduced side effects in comparison to prednisolone, which causes bone loss through microCT analysis, glucose dysregulation via blood sugar and insulin levels, immunosuppression via flow cytometry of splenocytes, and stunted growth.^11^^,^^12^ Both here and in prior studies of *mdx* mice, we find prednisolone treatment significantly stunts growth of mice, while vamorolone treatment has much less of an effect on growth.^11^ Clinical data now show this side effect is similarly dissociated by vamorolone in humans. Recently, in a randomized, double-blind, placebo-controlled, parallel clinical trial of vamorolone and prednisone in patients with DMD, prednisone caused a significant decrease in the height percentile of patients. The prednisone group was significantly different from the two vamorolone treatment groups, neither of which showed a decrease in height percentile. Other groups have shown that MR agonists—like prednisolone—induce anxiety whereas MR antagonists are not anxiogenic.^40^^,^^41^ Here, we also find that prednisolone increases anxiety in *bmx* mice in the elevated plus maze, whereas vamorolone avoids this side effect. Future studies should expand on these findings from our ten-week trial to simulate the long-term clinical application of steroids in patients with BMD in order to assay their impacts on additional safety measures and later-stage outcomes such as heart function. Currently, our findings are generally consistent with differential safety profiles seen in clinical trials of vamorolone vs. prednisone.^13^

Chronic glucocorticoids can cause weight gain, obesity, and cushingoid features in patients. However, we and others consistently find that pharmacological glucocorticoids decrease body weight in *mdx* mice.^11^^,^^36^^,^^42^^,^^43^^,^^44^^,^^45^ These effects may reflect dose-dependent differences between species. Higher therapeutic doses of pharmacological (including prednisone, deflazacort, dexamethasone, and triamcinolone) glucocorticoids in rodents cause decreased body weight associated with increased leptin.^42^^,^^46^^,^^47^ In contrast, low-dose physiological glucocorticoid supplementation causes increases in rodent weight and aspects of adiposity or obesity.^48^^,^^49^^,^^50^^,^^51^ Differences in the content or responses of brown fat in rodents, which helps protect against metabolic dysfunction, may contribute to species differences.^51^^,^^52^^,^^53^ In rodent studies, factors such as dose, brown or browning fat, room temperature, diet, sex, genotype, and obesity status have all been found to affect glucocorticoid impacts on weight, muscle, metabolism, or obesity.^47^^,^^48^^,^^52^^,^^54^^,^^55^^,^^56^^,^^57^ Moving forward, studies focusing on obesity and metabolism in the etiology and treatment of muscular dystrophy are warranted. As this field continues to emerge, these could shed more insight into the role of mass and obesity on dystrophic phenotypes of patients or animal models, as well as on the potential to dissociate the metabolic side effects of steroids.

Our findings may have implications for other muscle diseases that are characterized by reductions in dystrophin secondary to inflammation, such as myositis.^15^ Female-manifesting carriers of DMD have also shown reductions in dystrophin not justified by the proportion of normal/abnormal *DMD* genes in their muscle, similarly suggesting inflammation-mediated dystrophin reductions that might be responsive to vamorolone treatment.^58^ In addition to patients with BMD, vamorolone has clear potential to increase dystrophin levels in patients with DMD treated with exon-skipping antisense oligonucleotide drugs (eteplirsen, golodirsen, viltolarsen, and casimersen). Since the dystrophin 3′ UTR is conserved between BMD and exon-skipped DMD transcripts, they should both be subject to regulation by DTMs and upregulation by steroids. One study examining dystrophin protein levels in *mdx* mice treated with 2′-*O-*methyl phosphorothioate antisense oligonucleotides showed increased dystrophin with prednisolone co-treatment compared to mice only treated with antisense oligonucleotide.^36^ This suggests co-treatment with vamorolone may allow for further increases of dystrophin protein levels in *mdx* mice and patients with DMD treated with exon-skipping drugs without the harsh side effects of prednisolone. Future studies in *mdx* mice co-treated with vamorolone and exon-skipping oligonucleotides are warranted.

In conclusion, we demonstrate that vamorolone increases motor function, improves muscle histopathology, and increases dystrophin protein levels in *bmx* mice with improved safety versus a traditional corticosteroid. Shared molecular pathologies of muscle inflammation and a secondary downregulation of dystrophin are seen in other muscle diseases such as myositis, LGMD, and myocardial infarction.^59^^,^^60^^,^^61^ Our data suggest vamorolone can particularly benefit the BMD and DMD patient populations, as well as other diseases impacting muscle or heart.

### Limitations of the study

A limitation of this study is that here we have shown preclinical efficacy of drug treatments in the *bmx* mouse model of disease, but have not tested clinical efficacy in human patients with BMD. A clinical trial of vamorolone was recently completed in patients with DMD (clinicaltrials.gov ID: NCT03439670)^13^ for which vamorolone is currently awaiting a regulatory approval decision, and a clinical trial of vamorolone is currently being conducted in patients with BMD (Clinical Trials ID # NCT05166109) to address this. We hypothesize that dystrophin-targeting miRNAs are responsible for decreased levels of dystrophin protein, and that anti-inflammatory steroids increase dystrophin protein by reducing expression of these miRNAs; data here demonstrate an inverse correlation of these levels; to fully demonstrate causation, further studies are needed to expand upon these and previous studies.^14^ Future investigations should be conducted to further clarify this mechanism, to determine the extent to which it impacts dystrophin in human muscle diseases, and to identify specific miRNAs that can be therapeutically targeted to increase dystrophin.

## STAR★Methods

### Key resources table

**Table:** 

REAGENT or RESOURCE	SOURCE	IDENTIFIER
**Antibodies**		

Recombinant Anti-Dystrophin antibody	Abcam	Cat# ab154168; RRID:AB_2858227
Monoclonal Anti-Laminin-2 (alpha-2 chain) antibody produced in rat	Sigma-Aldrich	Cat# L0663, RRID:AB_477153
Anti-mouse IgM (mu-chain specific)-FITC antibody produced in goat	Sigma-Aldrich	Cat# F9259, RRID:AB_259799
Goat anti-rabbit IgG (H+L) Highly cross-adsorbed secondary antibody, Alexa Fluor 568	Thermo Fisher Scientific	Cat# A-11036, RRID:AB_10563566
Donkey anti-rat IgG (H+L) Highly cross-adsorbed secondary antibody, Alexa Fluor 488	Thermo Fisher Scientific	Cat# A-21208, RRID:AB_2535794
Goat anti-rat IgG (H+L) Cross-adsorbed secondary antibody, Alexa Fluor 647	Thermo Fisher Scientific	Cat# A-21247, RRID:AB_141778
Sarcomeric Alpha Actinin Antibody	Abcam	Cat # ab68167; RRID:AB_11157538
Anti-rabbit secondary kit	ProteinSimple	Cat# 042-206

**Chemicals, peptides, and recombinant proteins**		

Humco Cherry Compounding Syrup	Amazon	Cat# B0742MXCQC
Prednisolone	Sigma-Aldrich	Cat# P6004
Vamorolone	Reveragen	N/A
Paraformaldehyde	Electron Microscopy Sciences	Cat# 15712-S
Triton X-100	Sigma-Aldrich	Cat# X100
Bovine Serum Albumin	Sigma-Aldrich	Cat# A7906
ProLong Gold Antifade Mountant with DAPI	Thermo Fisher Scientific	Cat# P36931
Hematoxylin	Sigma-Aldrich	Cat# 51275
Eosin	Sigma-Aldrich	Cat# 119830
Permount	Electron Microscopy Sciences	Cat# 17986-01
Sirius Red Fast Green stain	Chondrex	Cat# 9046
UltraPure 0.5M EDTA, pH 8.0	Thermo Fisher Scientific	Cat# 15575020
Tris-HCl buffer, pH 6.8 (1M solution)	MBL International	Cat# JM-2106-100
cOmplete ULTRA Tablets, mini EASYpack Protease Inhibitor Cocktail	Sigma-Aldrich	Cat# 05892970001
PhosSTOP phosphatase	Sigma-Aldrich	Cat# 04906837001
Wes Capillary immunoassay 66-440 kDA Separation Module	ProteinSimple	Cat# SM-W008
TRIzol Reagent	Thermo Fisher Scientific	Cat# 15596026

**Critical commercial assays**		

High Capacity cDNA Reverse Transcriptase Kit	Thermo Fisher Scientific	Cat# 4368813

**Experimental models: Organisms/strains**		

*bmx* mice	Heier et al.^19^	N/A

**Oligonucleotides**		

See Table S1 for Taqman Primers		

**Software and algorithms**		

ImageJ	NIH	RRID:SCR_003070
GraphPad Prism	GraphPad	RRID:SCR_002798
Fiji	NIH	RRID:SCR_002285
MuscleJ	Mayeuf-Louchart et al.^62^	https://github.com/ADanckaert/MuscleJ
Colour Deconvolution 2 plugin	Landini et al.^63^	
Compass for Simple Western		RRID:SCR_022930

### Resource availability

#### Lead contact

Further information and requests for resources and reagents should be directed to and will be fulfilled by the lead contact, Christopher R. Heier (cheier@childrensnsational.org).

#### Materials availability

This study did not generate new unique reagents. Materials used or generated in this manuscript will be shared upon request upon completion of a material transfer agreement.

### Experimental model and subject details

#### Animals

All animal studies were done in adherence to the NIH Guide for the Care and Use of Laboratory Animals. All experiments were conducted according to protocols within the guidelines and under approval of the Institutional Animal Care and Use Committee of Children’s National Medical Center. Animals were maintained in a controlled mouse facility with a 12 h light: 12 h dark photoperiod, fed *ad libitum*, and monitored daily for health. *bmx* mice contain an endogenous deletion of murine dystrophin exons 45-47, are maintained on a C57/BL6J background, and were previously characterized.^19^ Only male mice were used given that BMD is an X-linked disease that almost exclusively affects males. Age of mice was 6 weeks at treatment initiation; treatments were administered for 10 weeks and mice were 16 weeks of age at trial conclusion.

### Method details

#### Drug treatment

Mice were randomly divided into age-matched and weight-matched groups (n = 12 mice per group), with subsequent phenotyping experiments blinded to treatment group. Mice were treated with either daily oral vehicle (cherry syrup), prednisolone (5 mg/kg), or vamorolone (20 mg/kg) starting at 6 weeks of age. At 16 weeks of age, mice were sacrificed, terminal assays performed and tissues collected for subsequent analyses. Functional testing and terminal assays were performed on all mice. Following sacrifice, subsequent molecular and histological experiments were performed on skeletal muscle from a subset of n = 8 mice per group; for this, mice were selected at random from within each treatment group for subsequent processing and experimental analyses. This sample size was found to be adequately powered to detect differences of treatment in prior molecular and histological studies of *mdx* muscular dystrophy mice.^11^^,^^12^

#### Motor function tests

Forelimb and Hindlimb grip strength was assessed using a grip strength meter (Columbus Instruments) daily for 5 consecutive days according to Treat NMD protocols (DMD_M.2.2.001), with data interpreted as averaged maximum daily values. Two-limb wire hang and four-limb grid hang tests were performed in accordance with Treat NMD protocols (DMD_M.2.1.005). For two-limb wire hang, a wire hanger was suspended placed ∼35cm above a cage with soft bedding. Mice were hung using only their forelimbs; however, they were allowed to swing and hang with all four limbs if able. Hang time was recorded, with 600 seconds used as a cutoff. For four-limb grid hang tests the same parameters were used (35cm elevation, 600 second cutoff), but mice instead hung upside down from a handmade box covered in wire mesh (1x1cm grid).

#### Elevated plus maze

At 12 weeks of age, mice were placed at the junction of the open and closed arms of the maze, facing the open arm opposite to where the experimenter is. The mouse was allowed to explore the elevated plus maze for 5 minutes while an overhead camera recorded the mouse’s movement. The total amount of time spent in the open vs. closed arms was quantified.

#### Immunofluorescence

Muscles were mounted on cork frozen in liquid nitrogen-cooled isopentane, and sectioned (8 μm) onto slides. For most immunofluorescence experiments, muscle sections were fixed in ice cold acetone for 10 minutes. For anti-IgM immunofluorescence, muscle sections were fixed with 4% paraformaldehyde for 10 minutes at room temperature. Slides were washed with 1X PBST (0.1% Tween 20), blocked for 1 hour (1X PBST with 0.1% Triton X-100, 1% BSA, 10% goat serum, and 10% horse serum), washed 3 times, then exposed to primary antibodies overnight at 4°C: anti-dystrophin 1:150 (Abcam, #ab154168), anti-laminin-2 1:100 (Sigma-Aldrich, Cat. #L0663), and anti-mouse IgM-FITC 1:100 (Sigma-Aldrich, #F9259). Secondary antibodies included: goat anti-rabbit 568 1:400 (ThermoFisher, #A-11036), donkey anti-rat 488 1:400 (ThermoFisher, #A-21208), and goat anti-rat 647 1:400 (ThermoFisher, #A-21247). Coverslips were mounted using Prolong Gold Mounting Medium with DAPI (ThermoFisher, #P36931). Slides were imaged using an Olympus VS-120 scanning microscope at 20X.

#### Histological staining

Hematoxylin and eosin was performed to analyze necrosis and inflammation. Briefly, tissue sections were stained for 10 minutes with hematoxylin and excess stain was removed in running tap water until nuclei turned blue. Slides were incubated in 70% ethanol for 3 minutes and then stained with eosin for 3 minutes. Tissue sections were dehydrated with ethanol and cleared with xylene. Coverslips were mounted with permount. Sirius red fast green staining was performed to analyze fibrosis. Briefly, tissue sections were fixed in 4% paraformaldehyde for 10 minutes followed by one wash in distilled water. Sections were incubated with dye solution (Chondrex #9046) for 30 minutes in a humidified chamber at room temperature. Tissue sections were rinsed with distilled water until the water ran clear and then successively dehydrated in 70, 95, and 100% ethanol. Slides were cleared with xylene and coverslips were mounted using permount. All slides were imaged using an Olympus VS-120 scanning microscope at 20X.

#### Muscle histology analysis

Cross-sectional area (CSA) and minimum Feret’s diameter were determined using the MuscleJ macro for Fiji.^62^ The variance coefficients for CSA and minimum Feret’s diameter were calculated by dividing standard deviation by the average and multiplying by 1000. Centrally nucleated fibers were counted manually in ImageJ. Percentage of IgM-positive myofibers was calculated to quantify muscle damage.

To analyze fibrosis, the Colour Deconvolution 2 plugin for ImageJ was used to separate the Sirius red and fast green dyes.^63^ The layer containing Sirius red staining was thresholded, the muscle section outlined, and percent area with Sirius red staining was quantified. Hematoxylin and Eosin staining was performed to assay necrosis and inflammation. ImageJ was used to outline areas of necrosis and inflammation.

#### Protein extraction and wes

Muscles were dissected and frozen in liquid-nitrogen cooled isopentane. 8μm sections were lysed in High SDS buffer containing 0.02% EDTA (pH 8.0), 0.075% Tris-HCl (pH 6.8), and protease and phosphatase inhibitors. Capillary Western immunoassay (Wes) analysis was performed according to manufacturer’s instructions using 66–440 kDa Separation Modules (ProteinSimple). In each capillary 0.2mg/mL protein was loaded for analysis with antibodies to dystrophin (Abcam #ab15277, dilution 1:15) or alpha-actinin (Abcam #ab68167, dilution 1:100), and anti-rabbit secondary (ProteinSimple #042-206). Compass for SW software was used to quantify chemiluminescence data, with data reported as % *bmx* + vehicle.

#### RNA extraction and qPCR analysis

qRT-PCR of miRNAs and mRNAs was performed as previously reported,^15^ with details and assay IDs provided in Supporting Materials (Table S1). ∼50-100 sections (8μm thickness) of mouse muscle were homogenized in 1 mL Trizol (Life Technologies) using a TissueRupter II homogenizer (Qiagen). RNA was converted to cDNA using the High Capacity cDNA Reverse Transcription Kit (Thermo Fisher #4368813).

### Quantification and statistical analysis

Statistical analyses were performed using GraphPad Prism v.9.0.0 (GraphPad Software, Inc.). The distribution of data points was assessed using the Shapiro-Wilk normality test and homogeneity of variances was assessed using a Brown-Forsythe test. Outliers were determined using a ROUT test where Q = 5%. Data were analyzed using a one-way analysis of variance (ANOVA; for comparisons with one independent variable and >2 groups). *Post hoc* analyses were performed using a Holm-Sidak test specifically comparing vehicle vs. prednisolone, and vehicle vs. vamorolone groups. A *p* value ≤ 0.05 was considered statistically significant. For all graphs, data are presented as mean ± SEM.

## Data Availability

• This study did not generate any large-scale sequencing or proteomics datasets. • This paper does not report original code or source code. • Any additional information required to reanalyze the data reported in this paper is available from the lead contact upon request.

## Supplemental information

Document S1. Figures S1–S5

Table S1. List of qRT-PCR assays used for gene expression analyses, related to STAR Methods
