# Supplementary material for: Vamorolone improves Becker muscular dystrophy and increases dystrophin protein in bmx model mice
Source: iScience. 2023 Jun 16;26(7):107161. doi: 10.1016/j.isci.2023.107161 (PMC10391915; doi:10.1016/j.isci.2023.107161)
Supplement: Document S1. Figures S1–S5 [file mmc1.pdf]

iScience, Volume *26*

Supplemental information

Vamorolone improves Becker muscular dystrophy and increases dystrophin protein in *bmx* model mice

Nikki M. McCormack, Nhu Y. Nguyen, Christopher B. Tully, Trinitee Oliver, Alyson A. Fiorillo, and Christopher R. Heier

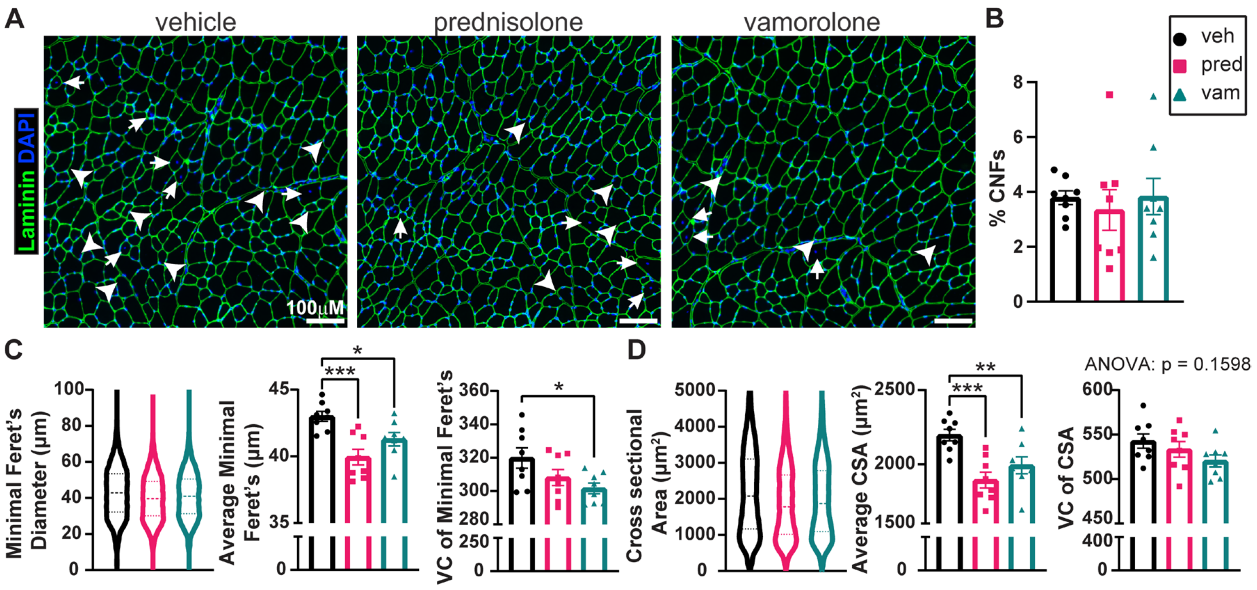

Figure S1. Vamorolone reduces myofiber size in the tibialis anterior, Related to Figure 3. (**A**) Tibialis anterior (TA) cross-sections were stained with laminin and DAPI to visualize myofiber membranes and nuclei. White arrows denote centrally nucleated fibers (CNFs). (**B**) The percentage of CNFs was unchanged in the TA with prednisolone and vamorolone. (**C**) Minimal Feret's diameter was determined for each myofiber and the variance coefficient (VC) was calculated. Variability of the minimal Feret's diameter was significantly reduced with vamorolone but not prednisolone. The average minimal Feret's diameter was significantly reduced with both treatments. (**D**) Cross-sectional area (CSA) was determined for each myofiber and the VC was calculated. Variability of the CSA was unchanged with prednisolone and vamorolone. The average CSA was significantly reduced with both treatments. n=8 per group. Data analyzed by one-way ANOVA followed by *post hoc* Holm-Sidak's multiple comparisons test. Data represented as mean ± S.E.M. ns p>0.05, *p≤0.05, **p≤0.01, ***p≤0.001.

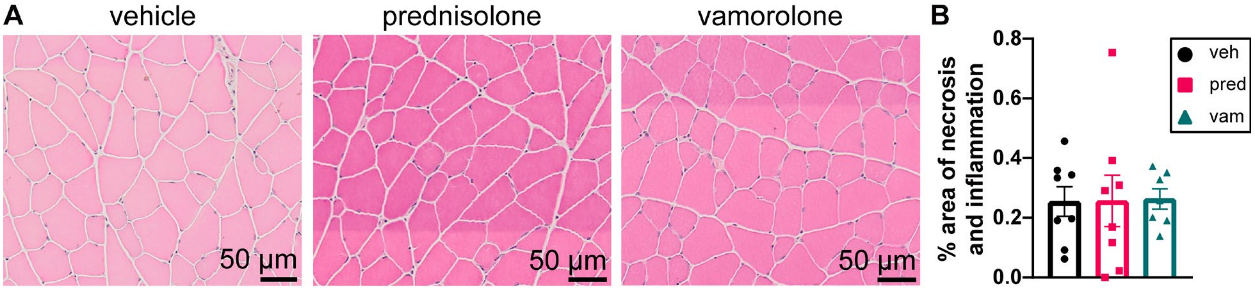

Figure S2. Treatment has no effect on necrosis and infiltration of immune cells, Related to Figure 4. (**A**) Hematoxylin and eosin staining of gastrocnemius muscle cross-sections. (**B**) The percentage of area of necrosis and inflammation was unchanged with prednisolone and vamorolone treatment.

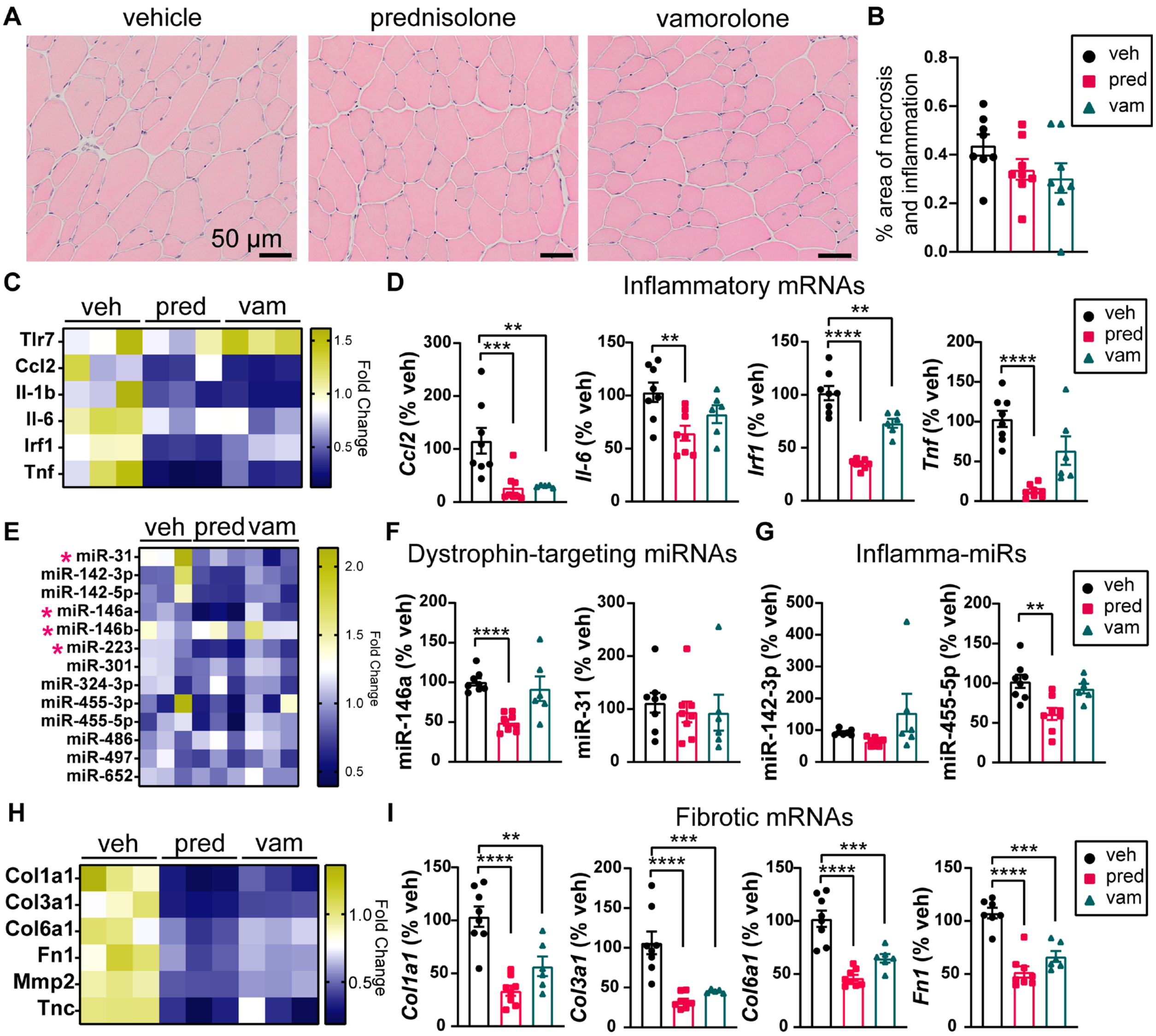

Figure S3. Vamorolone moderately reduces inflammation in the tibialis anterior, Related to Figure 4. (**A**) Hematoxylin and eosin staining of tibialis anterior (TA) cross-sections. (**B**) The percentage of area necrosis and inflammation was unchanged with prednisolone and vamorolone. (**C**) Heat map of inflammatory gene expression in the TA. (**D**) Graphs showing expression of inflammatory mRNAs. Expression of *Ccl2* and *Irf1* are significantly reduced with prednisolone and vamorolone. Expression of *Il-6* and *Tnf* are significantly reduced with prednisolone and modestly reduced with vamorolone. (**E**) Heat map of dystrophin-targeting microRNAs (DTMs) and inflamma-miRs in the TA. Pink asterisks denote DTMs. (**F**) Graphs

showing expression of DTMs in the TA. Expression of miR-146a and miR-223 are significantly reduced with prednisolone but not vamorolone. Expression of miR-146b and miR-31 are unchanged with treatment. (**G**) Graphs showing expression of inflamma-miRs in the TA. Expression of miR-497 was significantly reduced with prednisolone and vamorolone. miR-455-5p levels were significantly reduced with prednisolone but not vamorolone whereas expression of miR-142-3p and miR-142-5p were unchanged with treatment. (**H**) Heat map of fibrotic gene expression in the TA. (**I**) Graphs showing expression of fibrotic genes. Expression of *Col3a1* and *Fn1* are significantly reduced with prednisolone and vamorolone. n=8 per group. Data analyzed by one-way ANOVA followed by *post hoc* Holm-Sidak's multiple comparisons test. Data represented as mean ± S.E.M. ns $p>0.05$, **$p\leq0.01$, ***$p\leq0.001$, ****$p\leq0.0001$.

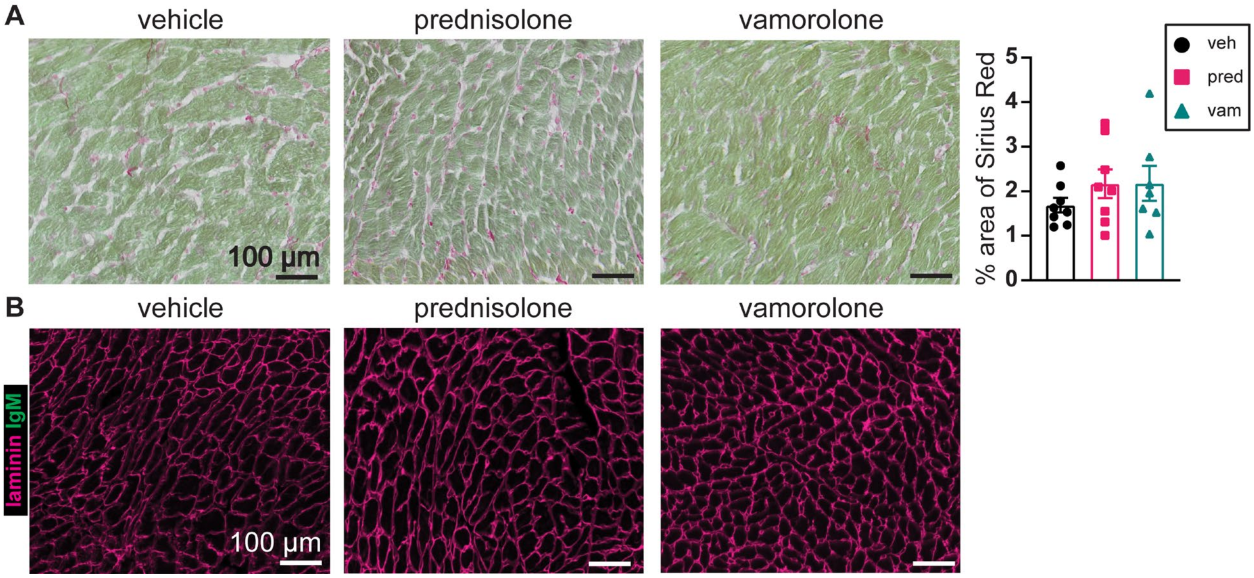

Figure S4. Treatment has no effect on cardiac fibrosis or damage, Related to Figure 5. (**A**) Representative images of Sirius red fast green (SRFG) staining in heart cross-sections and quantification of fibrotic area. Fibrotic area is unchanged with prednisolone and vamorolone treatment. (**B**) Representative images of heart cross-sections immunostained with laminin and IgM to visualize cardiac damage. No IgM+ staining was present in any mouse. n=8 per group.

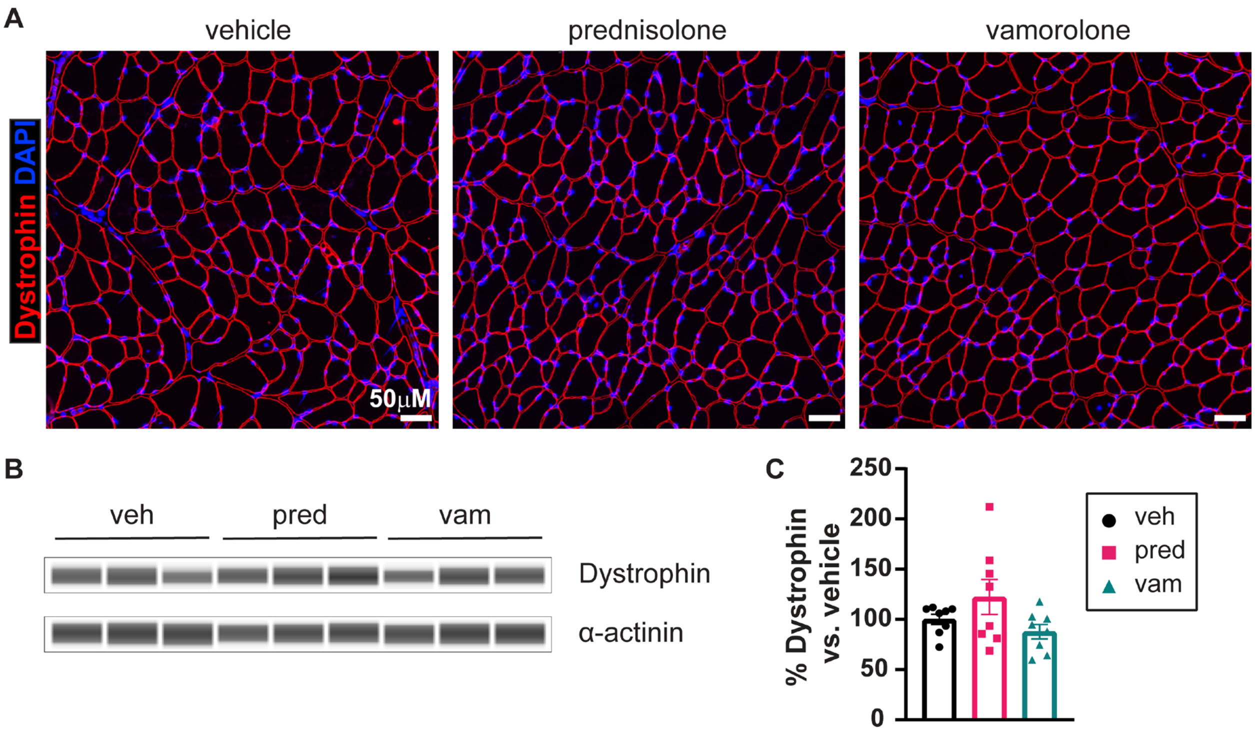

Figure S5. Vamorolone does not increase dystrophin protein levels in the tibialis anterior, Related to Figure 6. (**A**) Representative images of TA cross-sections immunostained with dystrophin. (**B-C**) Dystrophin protein levels were quantified by capillary electrophoresis (Wes). Dystrophin protein levels are unchanged in the TA with prednisolone and vamorolone treatment. n=8 per group. Data analyzed by one-way ANOVA followed by *post hoc* Holm-Sidak's multiple comparisons test. Data represented as mean ± S.E.M. ns $p>0.05$.
